# ID 251 - Custo-Efetividade e Análise de Impacto Orçamentário do Transplante Multivisceral em Pacientes com Tumores Abdominais, Catástrofes Abdominais e Tromboses Complexas

**DOI:** 10.5327/2237-9622.2025.v34s1.251

**Published:** 2025-11-25

**Authors:** Bruna Carolina de Araújo, Letícia Aparecida Lopes Bezerra da Silva, Roberta Crevelário de Melo, Cinthia Lanchotte Ferreira, Thaiana Helena Roma Santiago, Antonio Pescuma, Juliana Abud, Alessandra Crescenzi, Alexandre Chagas de Santana, Cláudia Lima Vieira, Inara Pereira da Cunha, Márcia Saldanha Kubrusly, Alex Jones Flores Cassenote, Wallace Breno Barbosa, Luciana Costa Xavier e Luciana Bertocco de Paiva Haddad

**Keywords:** transplante multivisceral, tumores abdominais, catástrofes abdominais, tromboses complexas, custo-efetividade, análise de impacto orçamentário

## Abstract

**Introdução:**

A prevalência de condições que indicam o transplante multivisceral (TMV) é relativamente baixa devido à complexidade e à gravidade das patologias que demandam essa intervenção. Exemplos dessas condições incluem tumores abdominais, desastres abdominais e tromboses difusas. O procedimento de TMV é altamente complexo e exige infraestrutura especializada e treinamento avançado. No entanto, ele pode ser a única alternativa terapêutica viável para pacientes em situações em que não existam outras opções de tratamento além do transplante. Nesse sentido, este estudo tem como objetivo avaliar a custo-efetividade e o impacto orçamentário na introdução do TMV.

**Método:**

Para a avaliação econômica, foi adotado o modelo de Markov, com quatro estados de saúde, e foram considerados os custos diretos do tratamento. O comparador utilizado foi o tratamento de suporte, uma vez que não há outra opção terapêutica disponível. A análise do impacto orçamentário foi feita sob a perspectiva do Sistema Único de Saúde (SUS), com um horizonte de cinco anos. Consideraram-se 15 pacientes por ano com indicação de TMV. Os cenários utilizados foram: cenário atual (lista de transplante, com taxa de manutenção de 0,5/ano e custo de R$ 600.000,00/ano); cenário 1 (TMV, com sobrevida em 5 anos de 0,62 a 0,47, e custo do procedimento de R$ 1.200.250,00, mais acompanhamento anual) e cenário 2 (custo do procedimento de R$ 402.417,79, com acompanhamento semelhante ao Cenário 1). A análise de sensibilidade determinística considerou variações de 20% em cada cenário. Os custos do procedimento se deram por meio dos convênios existentes.

**Resultados:**

A inclusão do TMV tem um custo incremental por paciente de R$ 2.962.082,67 para um horizonte temporal de 5 anos e a efetividade incremental de 2,39. A razão de custo efetividade incremental (RCEI) é de R$ 1.240.379,95. A análise de sensibilidade determinística demonstrou grande variação no custo do procedimento cirúrgico. Em relação à análise de impacto orçamentário, a incorporação do TMV gera um impacto incremental de R$ 20.133.750,00 a R$ 15.666.280,87 no cenário 1, totalizando R$ 87.431.032,35 em cinco anos. No cenário 2, o impacto é de R$ 8.166.266,85 a R$ 3.698.797,72, totalizando R$ 27.593.616,60. A análise de sensibilidade indicou custos variando de R$ 31.998.706,47 a R$ 191.992.238,82 no cenário 1, e de R$ 20.031.223,32 a R$ 120.187.339,92 no cenário 2.

**Conclusão:**

O TMV para as condições de tumores abdominais, catástrofes abdominais e tromboses complexas apresenta uma razão de custo incremental bastante elevada em relação aos comparadores, porém deve ser considerado num cenário de raridade da indicação e do acesso ao procedimento, devendo ser consideradas as imprecisões na avaliação do custo do procedimento. Observa-se que, na análise de impacto orçamentário, ambos os cenários apresentam custos maiores que o cenário atual nos primeiros cinco anos após o transplante. Contudo, dependendo dos valores do procedimento, os custos podem ser semelhantes ou menores em comparação ao cenário atual.

